# Mastering your fellowship: Part 1, 2026

**DOI:** 10.4102/safp.v68i1.6209

**Published:** 2026-01-19

**Authors:** Klaus B. von Pressentin, John M. Musonda, Joyce Musonda, Gert Marincowitz, Selvandran Rangiah

**Affiliations:** 1 Division of Family Medicine, Department of Family, Community and Emergency Care, Faculty of Health Sciences, University of Cape Town, Cape Town, South Africa; 2 Department of Family Medicine and Primary Care, Faculty of Health Sciences, University of the Witwatersrand, Johannesburg, South Africa; 3 Department of Family Medicine, Faculty of Health Sciences, University of Pretoria, Pretoria, South Africa; 4 Department of Family Medicine, Faculty of Health Sciences, University of Limpopo, Polokwane, South Africa; 5 Department of Family Medicine, College of Health Sciences, University of KwaZulu-Natal, Durban, South Africa

**Keywords:** family physicians, FCFP (SA) examination, family medicine registrars, postgraduate training, national exit examination, paediatrics, primary care

## Abstract

The series ‘Mastering your Fellowship’ provides examples of the question formats encountered in the written and clinical examinations, Part A of the FCFP (SA) examination. The series aims to help family medicine registrars (and supervisors) prepare for this examination. Model answers are available online.

This section in the *South African Family Practice* journal aims to help registrars prepare for the FCFP (SA) Final Part A examination (Fellowship of the College of Family Physicians). It will provide examples of the question formats encountered in the written exam: multiple choice question (MCQ) in the form of single best answer (SBA – Type A) and extended matching question (EMQ – Type R); short answer question (SAQ), questions based on the critical reading of a journal article (CRJ: evidence-based medicine) and an example of an objectively structured clinical examination (OSCE) question. Each of these question types is presented based on the College of Family Physicians blueprint and the key learning outcomes of the FCFP (SA) programme. The MCQs draw on the 10 clinical domains of family medicine, the SAQs align with the five national unit standards, and the critical reading section includes evidence-based medicine and primary care research methods.

This edition is based on entrustable professional activity (EPA) four (managing children with undifferentiated and more specific problems). We suggest that you attempt to answer the questions (either by yourself or with peers and supervisors) before finding the model answers online: http://www.safpj.co.za/.

Please visit the Colleges of Medicine website for guidelines on the Fellowship examination: https://cmsa.co.za/fellowship-of-the-college-of-family-physicians-of-south-africa-fcfpsa/. We are keen to hear how this series helps registrars and their supervisors prepare for the FCFP (SA) examination. Please email us your feedback and suggestions.

## Multiple choice question: Single best answer

A 9-month-old baby is brought to the district hospital with poor feeding, difficulty in breathing and a cough for the past 2 days. The mother reports no fever and no history of previous similar episodes. On examination, the infant’s temperature is 37.8°C, heart rate of 170 beats per minute (bpm), oxygen saturation of 86% on room air, capillary refill time of 3 s and body weight of 8.4 kg. Signs of respiratory distress are present: nasal flaring, subcostal recessions, grunting and a respiratory rate of 76 breaths per minute. On auscultation, there are inspiratory crackles and bilateral wheezing, with no signs of lobar consolidation. In addition to providing supplemental oxygen, what is the most appropriate next step in the management of this infant?

1. Administer nebulised salbutamol and prednisolone.
2. Keep the baby warm, start intravenous (IV) ceftriaxone and give fluids as needed.
3. Maintain hydration and monitor the infant for any deterioration.
4. Administer ipratropium bromide nebulisations and IV hydrocortisone.

Short answer: (3)

### Discussion

Based on the South African standard treatment and Integrated Management of Childhood Illnesses (IMCI) guidelines, the infant has severe acute bronchiolitis for which the mainstay of treatment is supportive care. The primary pathogen is the respiratory syncytial virus (RSV), a viral lower respiratory infection, and is the most common cause of wheezing in infants. A wheeze in a child under 1 year old, bilateral and with no prior episode, supports the diagnosis. In the management of hypoxia (SpO_2_ < 90%), low- or high-flow oxygen therapy is the primary treatment, depending on the availability and severity of the condition. As the infant’s oxygen saturation is 86% in room air, supplemental oxygen is already indicated (as mentioned in the question) to maintain adequate oxygenation.

Beyond oxygen, management includes ensuring that the infant is well-hydrated (via oral or IV fluids, if needed, as poor feeding is a common issue) and closely monitoring for any worsening of respiratory status. Supportive measures, such as feeding support, nasal suctioning of secretions and hydration, have been shown to aid infants with bronchiolitis in their recovery. In contrast, aggressive pharmacological treatments have had little impact.

The use of oral or IV steroids and bronchodilators, such as salbutamol and ipratropium, is not recommended for the routine treatment of acute bronchiolitis because they have no proven benefit in most infants. If the baby is unable to feed orally, IV fluids or nasogastric feeds are used to maintain hydration. Unless there is clear evidence of secondary bacterial infection, antibiotics are not indicated in the routine treatment of bronchiolitis. Antibiotics can be harmful and may lead to side effects or the development of antibiotic resistance.

It is essential to differentiate viral bronchiolitis, bacterial pneumonia and asthma. Bacterial pneumonia may present with fever and focal crepitations, while asthma typically presents with symptoms, which are often episodic and rare in children under 1 year old. In addition, asthma exhibits a bronchodilator response, which helps to alleviate bronchospasm and wheezing.

Further reading

- National Department of Health, South Africa. Standard treatment guidelines and essential medicines list – Hospital level (Paediatrics) [homepage on the Internet]. 2023 ed. Updated May 2025 [cited 2025 Jul 31]. Available from: https://www.health.gov.za/nhi-edp-stgs-eml/.
- National Department of Health, South Africa. Integrated management of childhood illness (IMCI) – Hospital care book [homepage on the Internet]. Update 2022 [cited 2025 Jul 31]. Available from: https://knowledgehub.health.gov.za/elibrary/integrated-management-childhood-illness-2022.
- Dangor Z, Lala SG, Verwey C, et al. Bronchiolitis v. bronchopneumonia: Navigating antibiotic use within the lower respiratory tract infection spectrum. South African Medical Journal. 2023;113(6):1199–1202.

## Short answer question: ‘Leadership and clinical governance’, in the clinical domain of ‘child health’

You are the family physician in a rural district hospital, 3 h by road from the nearest neonatal intensive care unit (ICU). An unbooked teenage mother presents in labour at term gestation. Your community service medical officer (CSMO) calls you at 02:00, about 1 h after delivery, to inform you that she was able to perform a breech delivery of a 3500 g baby with features that raise concerns about the potential risks for hypoxic-ischaemic encephalopathy (HIE) after delivery. The reason for the delay in contacting you is that the CSMO had to focus on stabilising the mother, who experienced postpartum haemorrhage because of an atonic uterus. They would like guidance on the appropriate management and referral of the newborn baby with suspected HIE. The colleague sounds very anxious over the phone.

This question was included in the Second Semester 2024 FCFP(SA) written paper.

1. Using a leadership framework, describe your immediate approach to this situation during the next hour. (5 marks)
2. The CSMO does not arrive for the handover round the following morning. You receive a short text message from her stating that she wants to take a break from medicine. Describe your approach to supporting her. (5 marks)
3. The medical officer (MO) working in the neonatal ICU phones you the following day and starts shouting in frustration about how the baby has severe HIE and intractable convulsions. He threatens to contact the referral hospital’s clinical manager to accuse you and the junior doctor of negligent practice. Describe your approach to managing this phone call. (5 marks)
4. In preparation for the perinatal mortality meeting the following month, the neonatal consultant sends an email to you and your clinical manager to inform you that he plans to discuss the ‘poor management’ of this baby who passed away because of HIE. Describe which principles you would highlight during the opening remarks as the chair of the mortality meeting. (5 marks)
5. Following the perinatal mortality meeting, your clinical manager calls you into her office to discuss your feedback on addressing the recommendations from the perinatal mortality meeting. The meeting recommended staff rostering (ensuring more senior doctors are on site) and reviewing the thresholds for requesting senior assistance. System challenges related to ambulance availability must be addressed, and there should be a renewed effort to ensure that teenage mothers have adequate access to antenatal care. Describe your approach to this conversation with the clinical manager to develop a shared understanding of who is responsible for addressing these recommendations. (5 marks)

Total: 25 marks

Suggested answers (the answers should show some application to the scenario).

1. **Using a leadership framework, describe your immediate approach to this situation during the next hour. (5 marks)**
  - The ‘I-we-it’ model of leadership applies. The model answer could also be applied to alternative leadership frameworks.
  - I: Emphasises personal leadership, self-awareness and self-management – ensure that you remain composed and calm to manage this stressful situation in the middle of the night, and the need to gauge whether you need to support telephonically or in person on site.
  - We: Leading through relationships and teams. Focus on supporting the junior colleague and the maternity team in managing this situation appropriately from here on. Gauge whether the junior colleague can safely manage this baby at risk of HIE and the need for cooling and transfer.
  - It: Engaging with systems and services. The clinical situation likely exceeds the local team’s capacity. There is a need to follow the local protocols and processes, both clinical (stabilising the baby and mom and commencing cooling protocol within 6 h) as well as the administrative tasks of transferring both baby and mom to the referral hospital, including the consideration of road versus air transfer (this is an after-hours emergency that will require transfer by road).
  - There is an overlap of leadership roles: consultant, clinical governance, and supervisor or capacity builder.
2. **The CSMO does not arrive for the handover round the following morning. You receive a short text message from her stating that she wants to take a break from medicine. Describe your approach to supporting her. (5 marks)**
  - Aim to contact the junior colleague and the night team to obtain collateral information, such as her state when she left (mark for obtaining collateral information).
  - Assess the current and previous mental health risks of burnout and offer support. This acute crisis could exacerbate an existing mental health condition.
  - Assess the colleague’s support network and intervene if there are none, such as regular check-ins.
  - Arrange a debriefing with her (and her team) and refer her for mental health support and counselling.
  - Review work arrangements and arrange roster swaps where possible.
3. **The MO working in the neonatal ICU phones you the following day and starts shouting in frustration about how the baby has severe HIE and intractable convulsions. He threatens to contact the referral hospital’s clinical manager to accuse you and the junior doctor of negligent practice. Describe your approach to managing this phone call. (5 marks)**
  Avoid reacting with a loud voice or similar emotions, such as anger. Try to defuse the tense situation by listening calmly. Aim to maintain a professional, respectful and collegial demeanour.
  Assess whether the colleague is open to reasoning and understanding the facts of the event from the local perspective.

If the conversation continues in an abusive manner:

1. Warn: Give the colleague the benefit of the doubt by politely letting them know you want to assist them, but that it is difficult to do so when they are speaking to you in that manner.
2. Repeat the warning: If that does not work, repeat what you said and ask him politely to stop using abusive language. For example, say, ‘As I said, I would like to help, but I am having trouble focusing on the problem when you speak that way. Please stop so we can work this out’.
3. Politely hang up: On the third strike, inform the caller that you are no longer the person who can assist them. Let the caller know you will hang up and have a manager or consultant call them. Before you hang up, say, ‘I am sorry, but I am going to hang up now’, and then do it.

Propose a follow-up conversation (telephonically or in person) and indicate that you will strive to obtain factual information on any queries or accusations he may have.

If you cannot resolve the matter, inform the colleague that you plan to involve the district hospital manager to resolve the situation and phone him back (or his line manager, such as the consultant).

4. **In preparation for the perinatal mortality meeting the following month, the neonatal consultant sends an email to you and your clinical manager to inform you that he plans to discuss the ‘poor management’ of this baby who passed away because of HIE. Describe which principles you would highlight during the opening remarks as the chair of the mortality meeting. (5 marks)**
  Chairing this meeting requires self-awareness, self-regulation and a professional approach, especially as it involves uncovering learning gaps and areas for improvement in the service.
  Remind attendees of the purpose of the perinatal mortality meeting: to review the facts of the scenario, identify modifiable factors according to the Perinatal Problem Identification Programme (PPIP) framework and make recommendations to strengthen the health service.
  Remind attendees of the importance of applying key clinical governance tools, including fishbone analysis and the 5 Whys approach. This may help structure the approach to uncover the root causes of the situation and formulate appropriate recommendations.
  It is possible that individual factors did not cause the situation, but rather underlying systemic deficiencies in staffing arrangements, equipment, access to neonatal ICU services within the short timeframe for head cooling in suspected HIE and barriers to accessing antenatal care for teenage mothers.
  Agree on the principles for managing the meeting interactions. When responding to hostile remarks, keep your reply brief, informative, friendly and firm. Avoid defensiveness, arguments, emotions or justifications. Remain conscious of the need to maintain and grow relationships at individual and collective levels.
5. **Following the perinatal mortality meeting, your clinical manager calls you into her office to discuss your feedback on addressing the recommendations from the perinatal mortality meeting. The meeting recommended staffing rostering (ensuring more senior doctors are on site) and reviewing the thresholds for calling for senior help. System challenges related to ambulance availability must be addressed, and there should be a renewed effort to ensure that teenage mothers have adequate access to antenatal care. Describe your approach to this conversation with the clinical manager to develop a shared understanding of who is responsible for addressing these recommendations. (5 marks)**
  Aim to remain professional and respectful of your colleague, the clinical manager. Ensure that the conversation remains focused on the facts of the situation and the relevant policies and frameworks for managing a potential medicolegal case scenario.
  Aim to resolve the role and task distribution in managing activities related to the medicolegal situation, communication with the family and clinical governance, including addressing the root causes of the incident and implementing recommendations from the meeting.
  Prepare the minutes of the agreed strategy and task distribution in a concise summary with agreed action points involving appropriate colleagues and structures in the local setting, such as the medical manager, quality assurance manager and patient safety committee.
  Maintain and grow the relationship and suggest a follow-up conversation to reflect on the process and outcome of the shared management strategy for this incident.
  This is an opportunity to include the medical manager as the overall line manager and facility head to clarify the roles of the clinical manager and family physician in addressing issues that span both clinical and corporate governance domains. Use this conversation to establish a shared understanding of how best to support one another, particularly where roles overlap.

Further reading

- Mash R, Blitz J, Malan Z, Von Pressentin K. Leadership and governance: Learning outcomes and competencies required of the family physician in the district health system. S Afr Fam Pract. 2016;58(6):232–235. https://doi.org/10.1080/20786190.2016.1148338.
- Raheja R. Telephone triage: Managing difficult calls. AnswerStat [serial online]. 2013 [cited 2025 Jul 31]. Available from: https://answerstat.com/article/managing-difficult-telephone-triage-calls/.
- Eddy B. A better way to respond to difficult emails and texts. Psychology Today [serial online] 2021 [cited 2025 Jul 31]. Available from: https://www.psychologytoday.com/za/blog/5-types-people-who-can-ruin-your-life/202112/better-way-respond-difficult-emails-and-texts.
- Centre for Maternal, Fetal, Newborn and Child Health Care Strategies. The perinatal problem identification programme. University of Pretoria [cited 2025 Jul 31]. Available from: https://www.up.ac.za/centre-for-maternal-fetal-newborn-and-child-healthcare/article/2871749/the-perinatal-problem-identification-programme.
- Nonyane R, Du Plessis E, Clase J. Identifying avoidable causes of perinatal deaths in a district hospital in Lesotho. Curationis. 2024;47(1):2497. https://doi.org/10.4102/curationis.v47i1.2497.

## Critical appraisal of research

Read the accompanying article carefully and answer the following questions. As far as possible, use your own words. Do not copy out chunks from the article. Be guided by the allocation of marks concerning the length of your responses.

Profitt LB, Bresick G, Rossouw L, Van Stormbroek B, Ras T, Von Pressentin K. Healthcare access for children in a low-income area in Cape Town: A mixed-methods case study. S Afr Fam Pract. 2023;65(4):a5754. https://doi.org/10.4102/safp.v65i1.5754.

Total: 30 marks

1. Assess the thoroughness of the article’s title. (4 marks)
2. Was the use of mixed methods appropriate for the research question? Support your answer. (3 marks)
3. Critically comment on the adequacy of the sample size (*n* = 62) used in the community survey. (4 marks)
4. Critically analyse the sampling strategy of the community survey described in the article. (3 marks)
5. Was the survey instrument valid and reliable? (3 marks)
6. Evaluate the sampling approach used in the qualitative component of the study. (2 marks)
7. Critically appraise the results concerning the study objectives, including whether the results will help locally. (4 marks)
8. List two advantages and two disadvantages of using nominal group technique (NGT) in a healthcare setting. (4 marks)
9. Critically analyse the limitations of the study as discussed by the authors. (3 marks)

Suggested answers

1. **Assess the thoroughness of the article’s title. (4 marks)**
  The model answer is guided using frameworks such as PICO (Population, Intervention, Comparison, Outcome) or PCC (Population, Concept, Context).
  - P: Population well described: children in a low-income area of Cape Town.
  - I: Area of interest: health access.
  - C: Mixed methods imply assessing/describing health access from various angles.
  - O: Implied that health access will be described/assessed.
2. **Was the use of mixed methods appropriate for the research question? Support your answer. (3 marks)**
  - A mixed-methods study design was appropriate for this question, as the researchers aimed to investigate barriers and solutions to healthcare access for children. This objective necessitated the use of:
    ■ Quantitative methods to evaluate participants’ help-seeking patterns and the frequency of the identified barriers.
    ■ Qualitative methods to explore the underlying reasons for the existence of these barriers.
    ■ The NGT to build consensus around recommendations to improve access.
  - Mixed-method research is used to achieve a comprehensive understanding of the research problem, approaching it from different angles. The NGT is then tasked with consolidating the information and reaching a consensus among experts.
3. **Critically comment on the adequacy of the sample size (*n* = 62) used in the community survey. (4 marks)**
  - The authors clearly explain how they calculated the sample. The sample size was calculated to be 62, representing the population of 40 000 (estimated to be 10 000 households) with an expected rate of 80% experiencing barriers, a margin of error (precision) of 10% and a 95% confidence interval.
  - They have chosen a larger margin of error (10%) than is usually used (5%), which resulted in a small sample.
    ■ In general, 10% is not acceptable, but in exploratory studies (as this study), it could be sufficient.
    ■ Considering the 10% margin of error, the community survey results are not generalisable on their own.
  - They did not argue clearly why they expected the experience of barriers to be 80% (making the sample size smaller).
  - By comparing the findings, it was found that 74% of participants reported experiencing barriers, but 89% still believed healthcare was accessible.
4. **Critically analyse the sampling strategy of the community survey described in the article. (3 marks)**
  - The authors used cluster sampling in each of the four quadrants in formal housing areas, government housing and informal areas, also seeking foreign nationals.
  - This strategy enhanced broad representation in the area.
  - Other than that, it is not clear how participants were sampled within the different clusters, such as convenience sampling, or how many per cluster.
5. **Was the survey instrument valid and reliable? (3 marks)**
  - The authors indicate that the questionnaire was adjusted from a WHO verbal autopsy questionnaire.
  - A pilot study was conducted with five participants (recommended to be 10% of the sample) and translated into the most commonly used languages.
  - It can be concluded that the questionnaire was validated.
6. **Evaluate the sampling approach used in the qualitative component of the study. (2 marks)**
  - The sampling framework was clearly demonstrated in Figure 1 of the article.
  - Only 11 caregivers participated in the qualitative phase of the study, reaching data saturation by the end of the interviews.
7. **Critically appraise the results concerning the study objectives, including whether the results will help locally. (4 marks)**
  - It will depend on the reader’s setting; it may be partially applicable in settings that differ significantly from the research setting.
  - It appears to be applicable in most low-income settings in South Africa.
  - The results apply to low-income urban areas that face similar healthcare challenges.
  - However, rural areas may present different issues, such as distance and staff shortages.
8. **List two advantages and two disadvantages of using nominal group technique in a healthcare setting. (4 marks)**
  - Advantages (any two).
    ■ Equal and inclusive participation: ensures that all voices are heard, particularly those of quieter or junior staff members. This is achieved through silent idea generation and by providing equal opportunities for sharing ideas.
    ■ Reduces groupthink and bias through independent idea generation and anonymous voting, minimising peer pressure and dominant voices.
    ■ Efficient structured decision-making: The process is time-focused, structured and typically completed in a single session, facilitating rapid consensus on protocols or care pathways.
    ■ Transparent and open prioritisation: Ranking ideas using numbers helps in making decisions based on facts. This approach allows for easy explanation and justification of choices.
    ■ Adaptable to virtual formats (vNGT): Now effective online, enabling broad, diverse participation, cost savings and flexibility in scheduling.
  - Disadvantages (any two)
    ■ Topic limitations: Best suited for addressing a single issue per session; less effective when multiple topics need exploration.
    ■ Time and resource demands: Sessions require careful planning and typically last 60–90 min, accompanied by skilled facilitation.
    ■ Limited spontaneous interaction: The structured format may hinder natural dialogue and reduce opportunities for emergent ideas.
    ■ Challenges with large groups: Effectiveness tends to drop with larger groups; ideally used with 5–10 participants or by subdividing larger teams.
    ■ Virtual-specific issues: Online sessions may face technological challenges, digital literacy limitations and time-zone coordination problems.
9. **Critically analyse the limitations of the study as discussed by the authors. (3 marks)**
  - The limitations of the qualitative leg of the study (reference to language barriers, lost files/contact details) were discussed.
  - The limitations of the NGT have also been discussed in much detail (participants in NGT, plus the effect of the COVID-19 pandemic restrictions).
  - However, little reference is made to the weaknesses of the quantitative leg of the study, for example, sample size and selection.

Further reading

- Riley-Bennett F, Russell L, Fisher R. An example of the adaptation of the Nominal Group Technique (NGT) to a virtual format (vNGT) within healthcare research. BMC Med Res Methodol. 2024;24(1):240. https://doi.org/10.1186/s12874-024-02362-8.
- Lee SH, Ten Cate O, Gottlieb M, et al. The use of virtual nominal groups in healthcare research: An extended scoping review. PLoS One. 2024;19(6):e0302437. https://doi.org/10.1371/journal.pone.0302437.
- McMillan SS, King M, Tully MP. How to use the nominal group and Delphi techniques. Int J Clin Pharm. 2016;38(3):655–662. https://doi.org/10.1007/s11096-016-0257-x.
- Harb SI, Tao L, Peláez S, Boruff J, Rice DB, Shrier I. Methodological options of the nominal group technique for survey item elicitation in health research: A scoping review. J Clin Epidemiol. 2021;139:140–148. https://doi.org/10.1016/j.jclinepi.2021.08.008.

## Objectively structured clinical examination station scenario

### Objective

This station tests the candidate’s ability to consult holistically with a caregiver of a child presenting with undifferentiated systemic symptoms.

### Type of station

Integrated consultation.

### Role players

Caregiver (grandmother) of a 5-year-old male child.

### Instructions for the candidate

- You are the family physician working at a rural district hospital outpatient department.
- The grandmother of a 5-year-old boy has brought him in because of a 4-week history of intermittent low-grade fever, poor weight gain, increasing fatigue, and a recent episode of nosebleed. He has missed school because of being ‘too tired’. She is worried he may have HIV or TB (tuberculosis), but also mentions that ‘the mother said someone bewitched the child’.
- Your task: please consult with the grandmother to obtain a relevant history and then discuss your assessment and negotiate a management plan with her.
- You do not need to examine this patient. All relevant examination findings will be provided.

#### Instructions for the examiner

- This is an integrated consultation station, where the candidate has 20 min.
- Familiarise yourself with the assessor guidelines, which detail the expected responses from the candidate.
- No marks are allocated. In the mark sheet, tick off one of the three responses for each competency listed in Table 1. Ensure you are clear on the criteria for judging a candidate’s competence in each area.

**TABLE 1:** Marking sheet for consultation station.

Competencies	Candidate’s rating		
	Not competent	Competent	Good
Establishes and maintains a good clinician–caregiver relationship			
Gathering information: history/examination/investigations			
Clinical reasoning			
Explanation and planning			

#### Guidance for examiners

- This station challenges the candidate to manage a potentially serious and undifferentiated problem in a child, with contextual and psychosocial complexity.
- A working definition of competent performance is when the candidate effectively completes the task within the allotted time in a manner that maintains patient safety, even though the execution may not be efficient and well-structured.
  ■ Not competent: patient safety is compromised (including ethically and legally), or the task is not completed.
  ■ Competent: the task is completed safely and effectively.
  ■ Good: in addition to displaying competence, the task is completed efficiently and in an empathic, patient-centred manner (acknowledges patient’s ideas, beliefs, expectations, concerns or fears).

1. **Establishes and maintains a good clinician–caregiver relationship:**
  - Competent candidate:
    ■ Introduces themselves clearly and respectfully.
    ■ Ensures privacy and confidentiality, even in the presence of a crowded clinic environment.
    ■ Shows verbal and non-verbal listening (nodding, paraphrasing).
    ■ Allows the grandmother to express her main concern and listens without interruption.
    ■ Uses simple, non-judgmental language to promote trust
    ■ Asks the grandmother what she is most worried about (‘What are you most concerned about with your grandchild?’).
    ■ Ensures the caregiver feels acknowledged, especially when raising spiritual or cultural concerns.
  - Good candidate:
    ■ Builds rapport rapidly through warmth, empathy and cultural humility.
    ■ Validates grandmother’s emotional distress (‘It must be hard to see him like this and not know what’s going on’.)
    ■ Engages collaboratively, empowering the caregiver by including her in the reasoning process.
    ■ Normalises her fears (e.g. ‘Many grandmothers worry when children lose weight like this. You’re not alone’.).
    ■ Establishes a plan for ongoing partnership, even if referrals are needed.
2. **Gathering information: history/examination/investigations**
  - Competent:
    ■ Gathers a thorough history of the presenting complaint: duration and pattern of fever, weight loss, energy levels, bleeding symptoms and appetite.
    ■ Screens for TB contacts, HIV exposure, recent travel or infections.
    ■ Reviews immunisation, school attendance, nutrition and developmental milestones.
    ■ Checks for medication use, allergies and family history of chronic illness (HIV, TB, malignancy).
    ■ Asks basic questions about household structure (e.g. ‘Who lives with the child?’).
    ■ Collects symptoms and red flags (e.g. bleeding, splenomegaly).
    ■ Identifies caregiver’s uncertainty and possible defaulted maternal antiretroviral therapy (ART).
    ■ Notes caregiver burden, grandparent-headed household and missed school.
  - Good:
    ■ Uses a systematic, yet flexible history-taking approach.
    ■ Explores psychosocial risk factors (e.g. orphanhood, neglect, domestic tension, food insecurity).
    ■ Gathers information about cultural health-seeking behaviour (e.g. traditional healer visits, beliefs around witchcraft).
    ■ Probes for functional impairment (e.g. ‘Is he still playing like before? Can he carry his schoolbag?’).
    ■ Screens for emotional and behavioural changes, caregiver’s coping ability and support system.
3. **Clinical reasoning**
  - Competent:
    ■ Identifies this as a case of a child with an undifferentiated chronic systemic illness.
    ■ Constructs a problem list: fever, fatigue, weight loss, easy bruising.
    ■ Forms a basic differential: TB, HIV, malnutrition, leukaemia, anaemia, possibly a neglected chronic disease.
    ■ Proposes appropriate investigations: Full Blood Count (FBC), smear, HIV test, erythrocyte sedimentation rate (ESR)/C-reactive protein (CRP), Urea and Electrolytes (U&E).
    ■ This is a symptomatic child requiring active TB testing and exclusion as opposed to TB screening (verbal checklist).
    ■ Plans urgent referral for admission as the child would benefit from inpatient care and work-up.
    ■ Prioritises malignancy and HIV, and recognises red flag symptoms.
    ■ Begins to consider the caregiver’s emotional needs.
    ■ Acknowledges limited diagnostic capacity and need for system-level coordination.
  - Good:
    ■ Prioritises a comprehensive differential, including malignancy (e.g. Acute Lymphoblastic Leukemia), extrapulmonary TB, chronic viral infection, anaemia of chronic disease and malnutrition with possible bleeding disorder.
    ■ Integrates the biopsychosocial-spiritual model, for example, fear of a ‘curse’ may lead to treatment delay, avoidance of hospital.
    ■ Justifies choices of investigations and anticipates barriers to follow-up.
    ■ Recognises need for coordinated multi-sectoral intervention and interprofessional team-based care (social worker, school, dietician, outreach team).
    ■ Considers malignancy, HIV, chronic infection and nutritional deficiency.
    ■ Identifies low caregiver health literacy and confusion about overlapping symptoms.
    ■ Recognises social disintegration (parent absent), caregiver fatigue, informal housing conditions and risk of delayed care because of access issues.
    ■ Understands the impact of trauma, fear and lack of maternal guidance.
    ■ Integrates poverty, disempowerment and superstition in rural health-seeking.
4. **Explanation and planning**
  - Competent:
    ■ Explains concerns in simple language (e.g. ‘We are concerned about some serious illnesses that can make children lose weight and get tired’.).
    ■ Outlines plan for urgent blood tests and X-ray and suggests HIV screening and TB testing.
    ■ Encourages questions and clarifies misconceptions gently.
    ■ Avoids technical jargon, ensures grandmother understands and agrees to the next steps.
    ■ Confirms grandmother’s understanding and aligns expectations.
    ■ Acknowledges resource limitations and referral needs.
  - Good:
    ■ Uses visual aids or metaphors to aid understanding (e.g. ‘When our bodies don’t have enough healthy blood cells, they get tired like a battery running flat’).
    ■ Gently reframes witchcraft fears into medical explanations without invalidating her beliefs.
    ■ Facilitates shared decision-making, discussing implications of each test, and seeks verbal and emotional consent.
    ■ Invites a follow-up plan, including options for counselling and ongoing support.
    ■ Builds caregiver confidence to advocate for the child’s needs.
    ■ Explains how public sector referrals work and empowers navigation of the health system.
5. **Management**
  - Competent:
    ■ Orders appropriate initial investigations (FBC, HIV, ESR, TB screening tests, Random Blood Sugar (RBS), smear).
    ■ Discussion with pediatric consultant as part of patient safety concern and refers urgently if indicated (e.g. unstable vitals, pancytopenia).
    ■ Arranges clear follow-up and prepares the family for potential admission.
    ■ Initiates nutritional support and screens for immunisation gaps.
    ■ Ensures medical safety and initiates basic social support.
  - Good:
    ■ Facilitates holistic care: links with social worker for potential grant applications such as the child support grant, counselling for caregiver burnout and TB contact tracing team.
    ■ Flags case to multi-disciplinary team (paediatric outreach, home visit, nutrition).
    ■ Empowers the caregiver to continue accessing services even if the diagnosis is delayed.
    ■ Documents a sensitive safety-net plan for re-attendance.
    ■ Reduces caregiver anxiety and supports shared resilience
    ■ Anticipates and mitigates socio-economic obstacles to care.

#### Role play – Instructions for actor (simulated caregiver)

- Name: Mrs GA.
- Age: 61 years.
- Language: English (basic), isiZulu (mother tongue – use when distressed or overwhelmed).
- Relationship to child: Maternal grandmother, legal guardian.
- Occupation: Pensioner (only income is Older Persons Grant or old-age pension).
- Living situation: Lives with the child in a one-room shack in a rural village with no running water and a pit latrine.
- Transport: Travelled 2 h by minibus taxi to the clinic; often struggles to attend follow-ups because of cost.
- Family context:
  ■ Daughter (child’s mother) is HIV-positive, defaulted on ART, lives far away and has minimal contact.
  ■ No knowledge of the father.
  ■ Grandmother is the sole caregiver and decision-maker for the child.
  ■ Relies on neighbours for food and support.

You have brought your 5-year-old grandson to the clinic because he has had low-grade fever, weight loss, weakness and recently had a nosebleed. You are deeply worried that something serious is wrong. Use the responses depicted in Table 2.

**TABLE 2:** Instructions for actor.

If asked:	Your response should reflect:
What brings you here today?	‘He’s been sick for weeks. He just… fades. He doesn’t play. He looks tired all the time.’ *(teary-eyed)*
What are you most concerned about?	‘I’m scared it’s the same thing his mother had. But also […] some people are saying there’s something not natural going on.’
	‘Someone has done something to him.’ *(Looks away, whispers when speaking of witchcraft)*
How is the child doing at home?	‘He can’t even carry his schoolbag. I have to carry him on my back sometimes […] and I’m not young anymore.’
Do you have help at home?	‘No. I cook, clean, fetch water, take care of him […] all by myself. I’m tired. I’m scared.’
Has he been tested for HIV and TB before?	‘No […] I’ve been too scared. What if he is? What if it’s my fault?’
If the candidate speaks judgmentally about ‘bewitching’	Withdraw emotionally, say: ‘Maybe I shouldn’t have said anything. You doctors don’t believe in these things.’
If the candidate is warm and respectful	Open up emotionally: ‘I’ve been so alone with this. Thank you for listening.’
If the candidate asks about school or play	‘He doesn’t go anymore. He just sleeps. The teacher said he needs to see a doctor.’
If asked about food or grants	‘Sometimes we only eat pap and tea. the pension grant doesn’t go far. I try to stretch it.’
If offered HIV or TB testing	Show fear and uncertainty: ‘I don’t know […] What if people find out? What if they think I failed him?’
If offered support and explained in simple terms	‘Okay […] If it helps him, I will do it. I just want him better.’ *(softens)*
If the candidate gives a clear plan and support	Become hopeful and grateful: ‘So […] you’ll help us? We’ll come back if you tell us when. I’ll do what you say.’

You feel overwhelmed, afraid and conflicted about possible causes. You think it could be HIV, TB or possibly that someone has bewitched the child. You have been told different things by neighbours, family and the church.

Only reveal if the candidate explores appropriately:

- You feel guilty that you did not bring Mpho sooner.
- You are afraid that doctors will judge your parenting or blame you for the child’s illness.
- You feel ashamed that you believe in traditional causes of illness and worry others will laugh at you.
- You are exhausted and occasionally think you are failing as a caregiver.
- You feel unsupported and isolated – your family does not help, and the local clinic is far away.

Only reveal these when the candidate has built trust and explored the psychosocial context empathetically.

- Maintain a worried, fatigued posture.
- Speak quietly and hesitantly at first, then gradually open up if the candidate builds rapport.
- Tear up or use a handkerchief when discussing fears about HIV or death.
- Revert to isiZulu for emotional emphasis (e.g. ‘*Ngiyesaba, mntanami*’ [I’m afraid, my child]).
- Show cultural conflict: ‘At church, they prayed for him. I thought it was getting better, but now […]’.
- If dismissed: become guarded and uncooperative.

#### Clinical findings of the child

General appearance:

- Thin, visibly fatigued child seated passively on the caregiver’s lap.
- Appears pale and subdued, minimal spontaneous interaction and no apparent respiratory distress.

Growth and nutrition:

- Weight-for-age: below the 3rd centile (–2.5 SD), crossing two major percentile lines in the last 3 months, based on the Road to Health book.
- Height-for-age: just under the 5th centile.
- Mid-upper arm circumference (MUAC): 11.5 cm (borderline moderate malnutrition).
- Appetite: reported to be poor. Refuses solids, drinks diluted juice or tea.

Vital signs:

- Temperature: 37.9°C (low-grade fever).
- Heart rate: 122 bpm (tachycardia).
- Respiratory rate: 24 breaths/min.
- Blood pressure: 88/56 mmHg.
- SpO^2^: 96% on room air.
- Capillary refill time: 2 s.

Skin:

- Pallor: present, notably in conjunctiva and palms.
- Bruising: scattered ecchymoses on both lower legs and arms.
- Petechiae: fine petechial rash over the chest and neck.
- No rashes, ulcers or signs of scabies or eczema.

Head, eyes and ear, nose and throat (HEENT):

- Head: no dysmorphic features, fontanelles closed.
- Eyes: mild conjunctival pallor, no icterus and no conjunctivitis.
- Mouth: mild gingival bleeding at lateral incisors; otherwise, pink and moist mucosa.
- Lymph nodes: no generalised lymphadenopathy.

Chest and respiratory:

- Chest wall symmetrical, no recession or use of accessory muscles.
- Auscultation: Normal air entry bilaterally, no wheezes, crackles or dullness.
- Percussion: Normal resonance throughout.

Cardiovascular:

- Apex beat: visible in the 5th intercostal space, left mid-clavicular line.
- Heart sounds: dual, no murmurs.
- Peripheral pulses: present and equal bilaterally.
- Capillary refill: normal.

Abdomen:

- Soft, non-tender.
- Liver: palpable 3 cm below the costal margin; smooth edge.
- Spleen: palpable 2 cm – 3 cm below the costal margin.
- No masses, no ascites, bowel sounds present and normal.

Musculoskeletal:

- No joint swelling, redness or deformities.
- Decreased spontaneous movement and lies still during examination.
- Muscle wasting is visible in the thighs and upper arms.
- No bony tenderness.

Neurological:

- Alert but lethargic.
- Oriented to the caregiver but not playful or interactive.
- Tone: normal.
- Reflexes: normal
- Gait: not assessed (child too weak to stand unaided).
- No neck stiffness, photophobia or signs of meningism.

Developmental and behavioural:

- Reported regression in play and interaction over the past month.
- Previously verbal and socially engaged but now withdrawn.
- Does not initiate play or speech during consultation.

Rectal and genital examination:

- Not done during this consultation (not indicated unless suspected abuse or pathology).

Side room tests:

- Random blood glucose: 5.3 mmol/L.
- Ward Hb: 7.5 g/dL.
- HIV status: unknown; mother reportedly HIV-positive.
- TB screening questions: yes to cough, yes to fever, yes to poor weight gain and no known household contact with active TB.

Further reading

- National Department of Health, Republic of South Africa. Management of tuberculosis in children and adolescents [homepage on the Internet]. 2024 [cited 2025 Jul 31]. Available from: https://knowledgehub.health.gov.za/elibrary/management-tuberculosis-children-and-adolescents.
- Govender I, Rangiah S, Kaswa R, Nzaumvila D. Malnutrition in children under the age of 5 years in a primary health care setting. S Afr Fam Pract. 2021;63(3):5337. https://doi.org/10.4102/safp.v63i1.5337.
- National Department of Health, South Africa. Standard treatment guidelines and essential medicines list – Hospital level (Paediatrics) [homepage on the Internet]. 2023 ed. Updated May 2025 [cited 2025 Jul 31]. Available from: https://www.health.gov.za/nhi-edp-stgs-eml/.
- National Department of Health, South Africa. Integrated management of childhood illness (IMCI) – Hospital care book [homepage on the Internet]. Update 2022 [cited 2025 Jul 31]. Available from: https://knowledgehub.health.gov.za/elibrary/integrated-management-childhood-illness-2022.

